# Computational Methodologies in Synthesis, Preparation and Application of Antimicrobial Polymers, Biomolecules, and Nanocomposites

**DOI:** 10.3390/polym16162320

**Published:** 2024-08-16

**Authors:** Iva Rezić, Maja Somogyi Škoc

**Affiliations:** 1 Department of Applied Chemistry, Faculty of Textile Technology, University of Zagreb, 10000 Zagreb, Croatia; 2 Department of Materials Testing, Faculty of Textile Technology, University of Zagreb, 10000 Zagreb, Croatia; maja.somogyi@ttf.hr

**Keywords:** computational methodologies, polymer, nanocomposite, biomolecules, antimicrobial

## Abstract

The design and optimization of antimicrobial materials (polymers, biomolecules, or nanocomposites) can be significantly advanced by computational methodologies like molecular dynamics (MD), which provide insights into the interactions and stability of the antimicrobial agents within the polymer matrix, and machine learning (ML) or design of experiment (DOE), which predicts and optimizes antimicrobial efficacy and material properties. These innovations not only enhance the efficiency of developing antimicrobial polymers but also enable the creation of materials with tailored properties to meet specific application needs, ensuring safety and longevity in their usage. Therefore, this paper will present the computational methodologies employed in the synthesis and application of antimicrobial polymers, biomolecules, and nanocomposites. By leveraging advanced computational techniques such as MD, ML, or DOE, significant advancements in the design and optimization of antimicrobial materials are achieved. A comprehensive review on recent progress, together with highlights of the most relevant methodologies’ contributions to state-of-the-art materials science will be discussed, as well as future directions in the field will be foreseen. Finally, future possibilities and opportunities will be derived from the current state-of-the-art methodologies, providing perspectives on the potential evolution of polymer science and engineering of novel materials.

## 1. Introduction

Antimicrobial materials, including polymers, biomolecules, and nanocomposites, are critical in addressing the challenges posed by microbial resistance and infections, which are the most relevant problems related to human health today. Antimicrobial polymers are novel materials engineered to inhibit the growth of microorganisms such as bacteria, fungi, and viruses. These polymers are increasingly vital in applications ranging from medical devices and healthcare products to food packaging and water treatment systems. Recently, due to the COVID-19 pandemic, the importance of developing such materials has become even more evident.

The antimicrobial properties can be intrinsic, where the polymer itself possesses antimicrobial activity, or extrinsic, where antimicrobial agents such as metal nanoparticles, quaternary ammonium compounds, or natural antimicrobial peptides are incorporated into the polymer matrix. By integrating these agents, the polymers can effectively disrupt microbial cell membranes, interfere with cellular metabolism, or inhibit DNA replication, leading to the death or inhibition of the microorganisms.

Traditional synthesis and optimization methods are often time-consuming and labor-intensive. However, computational methodologies like MD simulations, density functional theory (DFT), and ML offer efficient and innovative approaches to material design and optimization [1,2,3,4]. This paper aims to review these computational techniques’ roles in developing antimicrobial materials, illustrating their impact on synthesis, optimization, and practical applications.

## 2. Overview of Antimicrobial Materials

### 2.1. Antimicrobial Polymers

**Figure 1:**
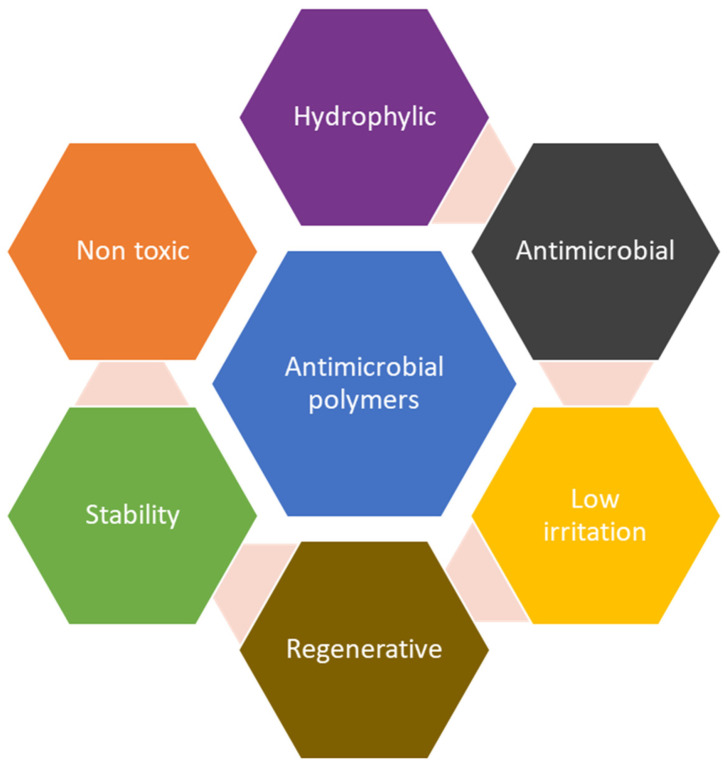
Desired properties of antimicrobial polymers.

Antimicrobial polymers are designed to inhibit the growth of microorganisms including bacteria, fungi, and viruses [1]. The broad spectrum of materials can have antimicrobial properties, but in order to be useful as functional antimicrobial materials, the polymers need to fulfill a number of other requirements, as presented in Figure 1.

Antimicrobial polymers have diverse applications, particularly in medical devices, healthcare products, food packaging, and water treatment systems, where maintaining hygiene and preventing infection are crucial. The antimicrobial properties of these polymers can be intrinsic, arising from the polymer’s inherent characteristics, or extrinsic, achieved by incorporating antimicrobial agents such as metal nanoparticles, quaternary ammonium compounds, or natural peptides into the polymer matrix [2]. These agents function by disrupting microbial cell membranes, inhibiting cellular processes, or damaging microbial DNA, effectively neutralizing the threat. Advances in computational methodologies, such as MD simulations and ML, have greatly improved the design and optimization of antimicrobial polymers. These technologies enable the precise modeling of polymer–agent interactions and the accurate prediction of material properties, leading to the development of more effective and tailored antimicrobial solutions. The broad applications of such materials require computational methods that can help in the prediction of polymer behavior and in the optimization of their antimicrobial properties [3].

Firstly, there are antimicrobial polymers with nanoparticles which, today, are a cutting-edge approach to combating microbial contamination and infection. By integrating nanoparticles such as silver, copper, and zinc oxide into polymer matrices, these materials harness the potent antimicrobial properties of the nanoparticles while maintaining the structural and functional benefits of the polymers. The nanoparticles work by releasing ions that disrupt microbial cell membranes, generate reactive oxygen species (ROS), or interfere with cellular functions, leading to the death of microorganisms. This integration not only enhances the antimicrobial efficacy but also provides sustained activity over time [4]. Applications of these advanced materials span across medical devices, wound dressings, textiles, food packaging, and water purification systems, where long-lasting antimicrobial protection is crucial. The development and optimization of antimicrobial polymers with nanoparticles have been significantly advanced by computational techniques such as MD simulations and density functional theory (DFT). These methodologies allow researchers to model the interactions between nanoparticles and polymer matrices at the atomic level, predict the behavior and efficacy of the composites, and optimize their properties for specific applications. This synergy between nanoparticles and polymers offers a robust platform for developing next-generation antimicrobial materials with enhanced performance and broad-spectrum activity.

Secondly, antimicrobial polymers incorporating quaternary ammonium compounds (QACs) offer an alternative yet effective strategy for controlling microbial growth in various environments. QACs are known for their potent antimicrobial activity, which stems from their ability to disrupt microbial cell membranes, leading to cell lysis and death. When integrated into polymer matrices, QACs provide durable and long-lasting antimicrobial protection, making them ideal for applications in healthcare, textiles, food packaging, and water treatment. The polymers act as carriers, enhancing the stability and controlled release of QACs thus maintaining their efficacy over extended periods. This integration also helps in reducing the potential for leaching and environmental contamination, ensuring that the antimicrobial action is localized and sustained. Advanced computational methodologies, such as MD simulations and ML, play a crucial role in optimizing the incorporation of QACs into polymers. These techniques allow researchers to predict the interaction between QACs and polymer chains, assess the stability and distribution of QACs within the polymer matrix, and fine-tune the material properties for maximum antimicrobial efficacy. As a result, antimicrobial polymers with QACs represent a versatile and powerful solution for enhancing hygiene and preventing microbial contamination across a broad spectrum of applications [5].

Thirdly, antimicrobial polymers incorporating natural peptides represent a promising and biocompatible approach to combating microbial infections. Natural antimicrobial peptides (AMPs) are small, naturally occurring proteins that have evolved to provide innate immune defense mechanisms in a wide variety of organisms. They are highly effective against a broad spectrum of microorganisms, including bacteria, fungi, and viruses, through mechanisms such as disrupting cell membranes, inhibiting cell wall synthesis, and interfering with intracellular functions [6]. When embedded within polymer matrices, these peptides retain their potent antimicrobial activity while gaining enhanced stability and prolonged efficacy. This combination is particularly valuable in medical applications such as wound dressings, surgical implants, and catheters, where preventing infection is critical. Additionally, these peptide-infused polymers can be used in food packaging to extend shelf life and in water purification systems to ensure safe drinking water. Advanced computational techniques, including MD simulations and bioinformatics, facilitate the design and optimization of these antimicrobial polymers by predicting peptide–polymer interactions, ensuring stability, and enhancing the controlled release of peptides. This innovative fusion of natural peptides with synthetic polymers offers a powerful, sustainable, and versatile solution to meet the growing demand for effective antimicrobial materials.

### 2.2. Antimicrobial Biomolecules

**Figure 2:**
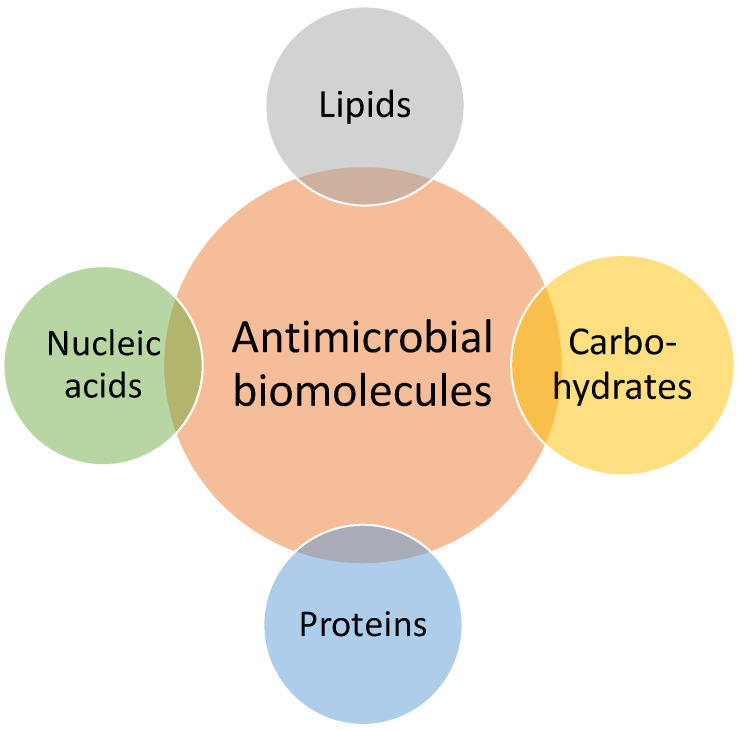
Antimicrobial biomolecules from different classes: nucleic acids, proteins, carbohydrates and lipids.

Antimicrobial biomolecules are a diverse group of naturally occurring or synthetically derived molecules that inhibit the growth of harmful microorganisms, including bacteria, fungi, and viruses. These biomolecules include antimicrobial peptides (AMPs), enzymes, nucleic acids, and small organic molecules, each with unique mechanisms of action (Figure 2).

Biomolecules such as antimicrobial peptides, enzymes, and nucleic acids play a significant role in combating microbial infections due to their diverse and potent mechanisms of action. Antimicrobial peptides (AMPs) disrupt microbial cell membranes, leading to cell lysis and death. Enzymes like lysozymes degrade bacterial cell walls, effectively neutralizing the bacteria. Nucleic acids, including RNA and DNA sequences, can interfere with microbial genetic material, inhibiting replication and transcription. Computational techniques are instrumental in understanding the mechanisms of these biomolecules and enhancing their stability and efficacy [7]. Through MD simulations, researchers can model the interactions between biomolecules and microbial targets at an atomic level, providing insights into their functional mechanisms. Additionally, ML algorithms can predict the antimicrobial activity of biomolecules based on their structure and properties, guiding the design of more effective compounds. Bioinformatics tools assist in identifying and optimizing nucleic acid sequences for targeted antimicrobial action. By leveraging these computational approaches, scientists can enhance the performance of antimicrobial biomolecules, making them more stable, potent, and suitable for various applications in medicine, agriculture, and biotechnology [8].

AMPs, for instance, disrupt microbial cell membranes, leading to cell lysis, while enzymes such as lysozymes degrade bacterial cell walls. Nucleic acids, including specific RNA molecules, can target and inhibit microbial genetic material, preventing replication and transcription. Small organic molecules can interfere with essential microbial metabolic pathways or cellular processes. The development and application of antimicrobial biomolecules are critical in various fields, including medicine, agriculture, and food safety, where they are used to prevent and treat infections, enhance crop protection, and preserve food products. Advances in computational methodologies, such as MD simulations, ML, and bioinformatics, have significantly enhanced the design, optimization, and understanding of antimicrobial biomolecules. These technologies allow for detailed modeling of biomolecule–target interactions, prediction of antimicrobial efficacy, and identification of potential modifications to improve stability and activity. By leveraging these computational tools, researchers can develop more effective and targeted antimicrobial therapies, addressing the growing challenge of microbial resistance and ensuring the safety and health of diverse environments [9].

### 2.3. Antimicrobial Nanocomposites

**Figure 3:**
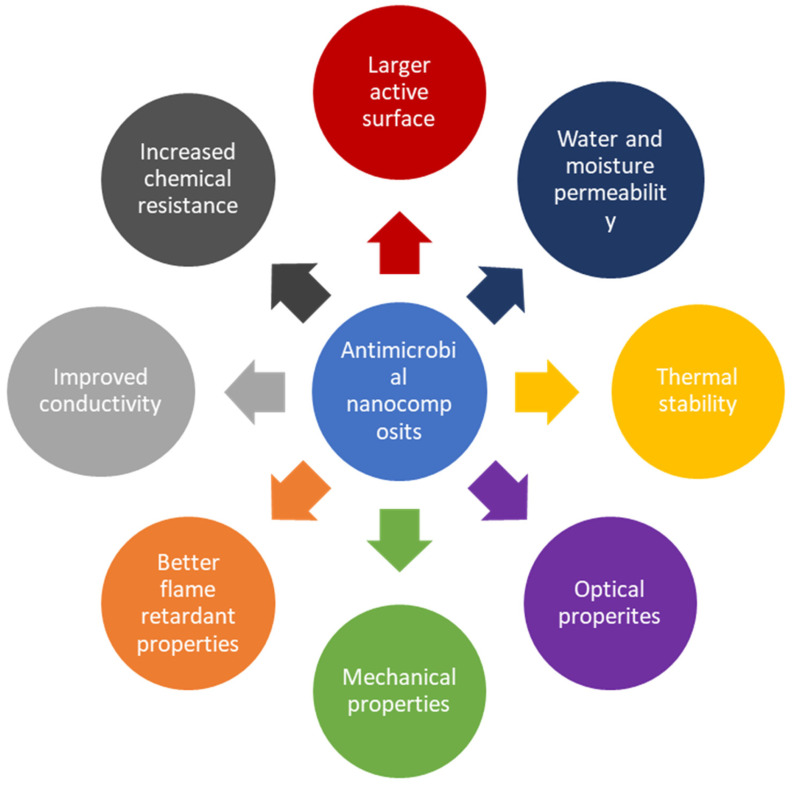
Antimicrobial nanocomposites with great high surface area and reactivity, increased chemical resistance, large active surface, excellent water and moisture permeability, thermal stability, conductivity as well as mechanical properties.

Nanocomposites integrate nanoparticles with antimicrobial properties into a matrix material, resulting in enhanced mechanical and antimicrobial performance. Computational tools aid in designing and optimizing these materials for various applications. Nanocomposites with antimicrobial properties are advanced materials that integrate antimicrobial agents, such as metal nanoparticles, within a composite matrix to achieve enhanced antimicrobial properties. These nanocomposites leverage the unique properties of nanoparticles, such as high surface area and reactivity, increased chemical resistance, large active surface, excellent water and moisture permeability, thermal stability, conductivity as well as mechanical properties needed to provide effective and sustained antimicrobial activity (Figure 3).

Common antimicrobial agents used in nanocomposites include silver, copper, zinc oxide, and titanium dioxide nanoparticles, which are known for their ability to disrupt microbial cell membranes, generate reactive oxygen species (ROS), and interfere with cellular functions, ultimately leading to microbial death. The incorporation of these nanoparticles into polymer matrices, ceramics, or other materials results in nanocomposites that are not only antimicrobial but also possess desirable mechanical, thermal, and chemical properties. This makes them suitable for a wide range of applications, including medical devices, food packaging, water purification systems, and surface coatings. For instance, silver nanoparticle-based nanocomposites are widely used in wound dressings and medical implants to prevent infections, while zinc oxide nanocomposites are utilized in sunscreens and coatings for their antimicrobial and UV-blocking properties [10].

Computational techniques play a crucial role in the design and optimization of antimicrobial nanocomposites. MD simulations help in understanding the interactions between nanoparticles and the composite matrix at the molecular level, ensuring uniform distribution and stability. Density functional theory (DFT) provides insights into the electronic properties and reactivity of nanoparticles, guiding the selection and functionalization of antimicrobial agents. ML algorithms can predict the performance of nanocomposites based on their composition and structure, optimizing their antimicrobial efficacy and material properties [11].

By combining these computational approaches, researchers can develop antimicrobial nanocomposites with tailored properties to meet specific application requirements, paving the way for innovative solutions to combat microbial contamination and infections across various industries [12].

## 3. Molecular Dynamics (MD) Simulations

MD simulations provide insights into the interactions and stability of antimicrobial agents within various matrices at an atomic level. Those simulations are a powerful computational technique used to study the physical movements of atoms and molecules over time [13]. By solving Newton’s equations of motion for a system of interacting particles, MD simulations provide detailed insights into the structural, dynamical, and thermodynamical properties of molecular systems. This method is particularly valuable in the fields of materials science, chemistry, biology, and biophysics, where understanding the behavior of complex molecular systems at an atomic level is crucial [14]. In the context of antimicrobial materials, MD simulations play a critical role in several key areas as follows: Interaction Studies: MD simulations allow researchers to model the interactions between antimicrobial agents (such as nanoparticles, peptides, or small molecules) and microbial cell components (like membranes and proteins). This helps in understanding how these agents disrupt microbial cell functions and cause cell death [15]. Process dynamic is monitored by simulating the behavior of antimicrobial agents within various matrices (such as polymers, biomolecules, or nanocomposites), MD simulations can predict their stability, distribution, and dynamics. This is crucial for optimizing the formulation and ensuring the sustained efficacy of antimicrobial materials [16].

Moreover, it is vital in material antimicrobial polymer design as MD simulations help in the rational design of new antimicrobial materials by predicting how modifications at the molecular level will affect the overall properties and performance of the material. For example, researchers can test different functional groups on nanoparticles or peptides to enhance their antimicrobial activity or compatibility with the matrix [17].

After functionalization, the MD predicts the mechanistic insights. It is enabled through detailed atomic-level simulations, during which the MD provides mechanistic insights into the modes of action of antimicrobial agents. This includes understanding how antimicrobial peptides penetrate and disrupt microbial membranes, how nanoparticles generate reactive oxygen species, or how small molecules inhibit key microbial enzymes [18]. Lastly, the simulations using MD assist in the optimization of the synthesis and processing conditions for antimicrobial materials. By simulating various conditions, researchers can identify the optimal parameters that lead to the best performance, reducing the need for extensive experimental trials. Overall, MD simulations are an indispensable tool in the development and optimization of antimicrobial materials, providing a deep understanding of molecular interactions and guiding the design of more effective and robust antimicrobial solutions [19].

### 3.1. Polymer–Nanoparticle Interactions

MD simulations help predict the compatibility and distribution of nanoparticles within polymer matrices, optimizing their antimicrobial properties and mechanical stability. Polymer–nanoparticle interactions are fundamental to the design and performance of nanocomposite materials, particularly in the context of antimicrobial applications. These interactions play a crucial role in determining the dispersion, stability, and overall properties of the nanocomposite. Understanding and controlling these interactions are essential for optimizing antimicrobial efficacy and ensuring the material’s integrity [20].

In polymer–nanoparticle interactions, process parameters are vital, so their optimization is crucial for obtaining the desired properties. Process parameters that can be optimized are as follows: (i) Dispersion: Achieving uniform dispersion of nanoparticles within the polymer matrix is essential for maximizing antimicrobial activity. Poor dispersion can lead to agglomeration, reducing surface area and limiting contact between nanoparticles and microbial targets. MD simulations provide insights into the thermodynamics and kinetics of nanoparticle dispersion within the polymer matrix, guiding the strategies to improve dispersion through surface modification or processing conditions [21]. (ii) Interface: The interface between nanoparticles and polymer chains significantly influences the overall properties of the nanocomposite. By altering the surface chemistry of nanoparticles or introducing compatibilizers, researchers can tailor the interactions at the interface to enhance compatibility, adhesion, and mechanical properties. Computational techniques, such as density functional theory (DFT), help in predicting and optimizing the interactions between nanoparticle surfaces and polymer functional groups [22]. (iii) Stability: The stability of the polymer–nanoparticle interface is crucial for long-term performance and durability. Weak interactions may lead to nanoparticle leaching or migration, compromising antimicrobial efficacy and material integrity. MD simulations enable the study of nanoparticle–polymer interactions under different environmental conditions, providing insights into factors affecting stability, such as temperature, pH, and mechanical stress. (iv) Antimicrobial mechanisms: The nature of polymer–nanoparticle interactions can influence the antimicrobial mechanisms of the nanocomposite. For example, nanoparticles may release antimicrobial ions or generate reactive oxygen species (ROS) upon interaction with microbial targets. Computational modeling helps in elucidating these mechanisms by simulating nanoparticle–cell interactions and predicting antimicrobial activity based on the extent of nanoparticle dispersion and surface coverage [23].

Therefore, by gaining a deeper understanding of polymer–nanoparticle interactions through computational modeling and experimental validation, researchers can optimize the design and performance of antimicrobial nanocomposites for various applications, ranging from medical devices to food packaging and water treatment [24].

### 3.2. Biomolecular Dynamics

Simulations aid in understanding the interaction of antimicrobial biomolecules with target microbes, shedding light on their mechanisms of action. Biomolecular dynamics refer to the study of the movements and interactions of biological molecules, such as proteins, nucleic acids, lipids, and small metabolites, within their environments. Understanding these dynamics is crucial for unraveling the mechanisms underlying biological processes, including enzyme function, signal transduction, molecular recognition, and membrane transport. Computational techniques, particularly MD simulations, play a pivotal role in exploring these complex phenomena at atomic and molecular levels [25].

They can be applied in the following areas:

(A) Protein Dynamics: Proteins are dynamic entities that undergo conformational changes essential for their function. MD simulations provide insights into these conformational changes, enabling the study of protein folding, ligand binding, enzyme catalysis, and allosteric regulation. By simulating the time evolution of protein structures, researchers can identify transient states and pathways that are not easily accessible through experimental techniques [26]. Nucleic Acid Dynamics: DNA and RNA molecules exhibit significant structural flexibility, which is crucial for processes such as replication, transcription, and translation. MD simulations help in understanding how nucleic acids interact with proteins, small molecules, and other nucleic acids. They also reveal the dynamics of secondary and tertiary structures, such as DNA helix melting, RNA folding, and ribosome function.
(B) Membrane Dynamics: Biological membranes are complex assemblies of lipids, proteins, and carbohydrates. MD simulations allow for the study of membrane organization, lipid–lipid and lipid–protein interactions, and the dynamics of membrane-bound proteins. These simulations are vital for understanding membrane permeability, signaling, and transport mechanisms. Enzyme–Substrate Interactions: Enzymes facilitate biochemical reactions by stabilizing transition states and lowering activation energy. MD simulations provide detailed views of enzyme–substrate interactions, revealing the dynamic process of substrate binding, product formation, and enzyme conformational changes. This information is critical for drug design and enzyme engineering [27].
(C) Molecular Recognition: Biomolecular interactions, such as protein–protein, protein–ligand, and protein–DNA interactions, are fundamental to cellular functions. MD simulations help elucidate the principles of molecular recognition, including binding affinity, specificity, and induced fit mechanisms. Understanding these interactions is essential for designing therapeutic agents and synthetic biomolecules [28]. Pathways and Mechanisms: MD simulations enable the exploration of dynamic pathways and mechanisms of biological processes. For example, they can simulate the entire catalytic cycle of an enzyme or the translocation of a substrate across a membrane. These simulations provide a comprehensive picture of the molecular events leading to biological outcomes.

Therefore, the applications of biomolecular dynamics in drug discovery are important since MD simulations are extensively used in drug discovery to predict the binding modes and affinities of drug candidates. They help in identifying potential binding sites, optimizing lead compounds, and understanding drug resistance mechanisms [29].

Moreover, the application in structural biology enables the complementing experimental techniques such as X-ray crystallography and NMR spectroscopy, while MD simulations offer dynamic views of biomolecular structures. They help in refining experimental models and interpreting structural data in the context of functional dynamics [30].

Additionally, in biomaterial design, an understanding of the dynamics of biomolecules is crucial for designing biomaterials with specific properties [31,32]. For instance, the dynamics of peptide-based hydrogels or DNA origami structures can be studied to optimize their stability and functionality. Synthetic biology also benefits from MD simulations due to the assistance in designing synthetic biomolecules and pathways by predicting the behavior of engineered proteins and nucleic acids. This aids in constructing biological systems with desired properties for applications in biotechnology and medicine. In summary, biomolecular dynamics provide a comprehensive understanding of the intricate movements and interactions of biological molecules. Computational techniques, especially MD simulations, are indispensable tools in this field, offering detailed insights that drive advancements in biomedical research, drug development, and the design of novel biomaterials.

## 4. Machine Learning (ML)

Machine learning techniques enable the prediction and optimization of material properties and their performance within different matrices. ML is a transformative technology with wide-ranging applications across various industries. Its ability to learn from data and make informed predictions or decisions makes it invaluable for solving complex problems and driving innovation. As computational techniques and data availability continue to advance, ML’s impact and potential will only grow, offering new opportunities and challenges for researchers and practitioners alike [32].

**Figure 4:**
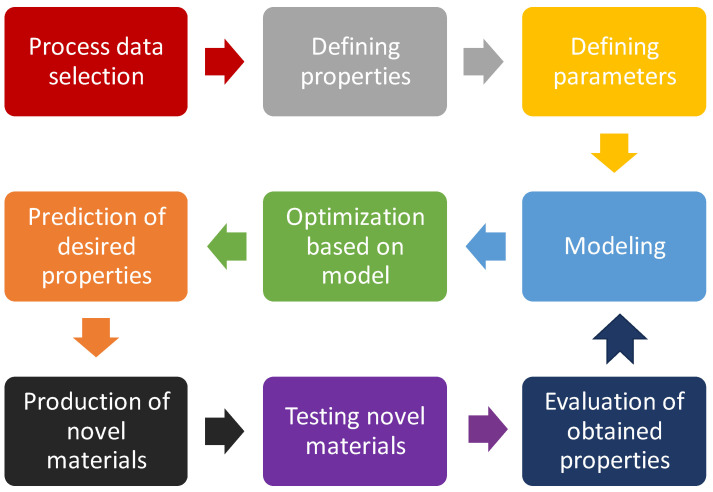
Process of development of novel material by using computational methodology such is ML or design of experiment, or others.

Advances in computational methods, such as MD simulations and ML, are increasingly used to predict and optimize the interactions and properties of nanocomposites, leading to the development of materials with tailored functionalities for diverse applications. Many different computational methods can be applied in designing, processing, and functionalization of novel materials. The schematic overview of this process is shown in Figure 4.

ML is a subset of artificial intelligence (AI) that involves the development of algorithms and statistical models, enabling computers to learn from and make predictions or decisions based on data. Unlike traditional programming, where specific instructions are coded, ML algorithms improve their performance on a task through experience, identifying patterns and relationships in data to make informed predictions or decisions [33].

In supervised learning, the algorithm is trained on a labeled dataset, where each training example is paired with an output label. The goal is to learn a mapping from inputs to outputs that can be generalized to unseen data [34]. Common supervised learning tasks include classification and regression. Unsupervised learning involves training on data without labeled responses. The algorithm seeks to identify underlying patterns or structures in the data. Common tasks include clustering and dimensionality reduction (e.g., principal component analysis) [35].

In reinforcement learning, an agent learns to make decisions by performing actions in an environment to maximize cumulative reward. It involves a feedback loop where the agent receives rewards or penalties based on its actions (e.g., game playing, robotic control). A subset of ML, deep learning involves neural networks with many layers (deep neural networks). These networks excel at learning hierarchical representations from raw data, enabling breakthroughs in image and speech recognition, natural language processing, and more.

### 4.1. Property Prediction by Deep Learning

ML models can predict the antimicrobial efficacy, stability, and toxicity of materials based on their physicochemical properties and synthesis conditions [36]. ML and deep learning (DL) techniques are proving to be a powerful tool in predicting the properties of drugs or antimicrobial polymer materials, significantly enhancing the ability to design materials with targeted antimicrobial properties without extensive trial-and-error experiments [37]. By leveraging vast datasets of polymer structures and their antimicrobial activity, ML algorithms can uncover complex relationships and predict the efficacy of new polymer candidates thus accelerating the development of antimicrobial materials [38]. ML algorithms are trained on labeled datasets to predict specific properties [39]. Common models include linear regression, support vector machines (SVM), decision trees, random forests, and neural networks. Unsupervised learning techniques like clustering and principal component analysis (PCA) can identify patterns and group polymers with similar antimicrobial properties [40,41,42,43,44,45,46,47,48,49,50,51,52,53]. Deep learning advanced models like convolutional neural networks (CNNs) and recurrent neural networks (RNNs) can process complex, high-dimensional data to predict antimicrobial activity from polymer structures.

The model can be evaluated through cross-evaluation, checking the performance metrics, but in the end, the most important is to test it with real data, which were not applied during the model development and optimization. This cross-validation is used to ensure the robustness of the model by dividing the dataset into training and testing subsets multiple times. Metrics such as mean absolute error (MAE), root mean square error (RMSE), accuracy, precision, recall, and the area under the receiver operating characteristic curve (AUC-ROC) are used to evaluate model performance. Through this, the ML model can predict the antimicrobial efficacy of polymers against various pathogens, including bacteria, viruses, and fungi. This helps in identifying promising candidates for medical and environmental applications.

Developing models that generalize well to new, unseen polymers and conditions is a key challenge. Techniques like transfer learning and domain adaptation are being explored to improve generalization. Combining ML predictions with experimental validation creates a synergistic approach, where ML guides experimental design and experimental results refine and improve ML models.

ML holds great potential for advancing the field of antimicrobial polymers by enabling accurate and efficient property prediction. By analyzing large datasets and uncovering complex relationships between polymer structures and antimicrobial activity, ML can accelerate the discovery and optimization of new antimicrobial materials. Despite challenges related to data quality, feature selection, and model interpretability, ongoing advancements in ML techniques and data integration are driving significant progress. As a result, ML is poised to play an increasingly important role in the development of innovative antimicrobial polymers for various applications, from healthcare to environmental protection.

### 4.2. Optimization of Synthesis Parameters

Algorithms optimize synthesis parameters to achieve materials with desired properties, reducing the need for extensive experimental trials [54,55,56,57,58,59]. ML is transforming the optimization of polymer synthesis parameters by enabling more efficient and accurate predictions of how variations in synthesis conditions affect polymer properties [60]. By leveraging vast datasets and sophisticated algorithms, ML can identify complex relationships between synthesis parameters and resultant polymer characteristics thus facilitating the design of polymers with desired properties through optimized synthesis processes. It is playing an increasingly important role in optimizing polymer synthesis parameters, offering a powerful approach to understanding and predicting how changes in synthesis conditions affect polymer properties. By leveraging advanced algorithms and extensive datasets, ML can uncover complex relationships and guide the design of polymers with tailored characteristics [61,62].

Despite challenges related to data quality, feature selection, and model generalization, ongoing advancements in ML techniques and data integration are driving significant progress [63,64,65]. As a result, ML is poised to enhance the efficiency and effectiveness of polymer synthesis, enabling the rapid development of innovative materials for a wide range of applications.

### 4.3. Integration in Composite Materials

ML assists in optimizing the integration of antimicrobial agents within composite materials, ensuring uniform distribution and enhanced performance. ML assists in optimizing the integration of antimicrobial agents within composite materials by ensuring uniform distribution and enhanced performance [66,67]. This advanced computational approach can significantly streamline the process of developing antimicrobial composites by predicting the optimal conditions for incorporating antimicrobial agents and foreseeing the resultant material properties. By leveraging advanced data analysis, predictive modeling, and optimization techniques, ML ensures uniform distribution and enhances the performance of antimicrobial composites [68,69]. This integration of ML in materials science accelerates the development of high-performance antimicrobial composites, offering promising applications in healthcare, packaging, and other industries where antimicrobial properties are critical.

## 5. Design of Experiment

Design of Experiment (DOE) is a systematic approach employed in scientific research to plan, conduct, analyze, and interpret controlled tests that evaluate the factors affecting a process and their interactions. In the realm of materials science and engineering, DOE is particularly invaluable as it enables researchers to optimize processes and product performance with a minimal number of experiments, thereby saving time and resources. By strategically varying input parameters and analyzing the resulting data, DOE helps in identifying the key factors and their optimal levels that contribute to desired outcomes. This method also provides insights into the interactions between different factors, which can be crucial for complex systems where multiple variables are at play. For instance, in the optimization of sol–gel processes for surface modification of polymers, DOE can be used to determine the best combination of precursor concentration, catalyst amount, and processing conditions to achieve the highest quality coating. Through the application of statistical tools such as response surface methodology (RSM) and factorial design, DOE enhances the robustness and reproducibility of experimental findings, facilitating the development of innovative materials and processes.

Design of Experiment (DOE) was already proven to be a powerful statistical tool, used for optimizing formulations, enhancing antimicrobial activity, and refining sol–gel processes, which we have efficiently used and reported in the literature [70,71,72,73,74,75]. Firstly, we used it in the optimization of formulations of detergent products in which the DOE was employed to identify the best combination of ingredients and processing conditions to achieve desired properties. Secondly, we applied it in the formulation of antimicrobial mixture of nanoparticles during the development of antimicrobial coatings, and optimized various processing factors such as the type of antimicrobial agent, concentration, solvent, and curing conditions by using DOE. Thirdly, we efficiently optimized the hydrophobicity of the surface of the material, enhancing the efficiency of the industrial process by 95%.

In all the cases, we combined the response surface methodology (RSM) to DOE for the optimization and prediction of the complex system. RSM is a more advanced DOE technique used for modeling and analyzing problems in which a response of interest is influenced by several variables. It involves fitting a polynomial equation to the experimental data and finding the optimal conditions for the desired response. For example, optimizing the release rate of an antimicrobial agent from a polymer matrix can be effectively achieved using RSM [70].

RSM followed screening experiments that were used to identify the most significant factors affecting antimicrobial activity. The design applied for modeling was a central composite design (CCD) for the fine-tuning of the levels of significant factors identified during screening [71]. By including axial points and center points, CCD provided a more detailed understanding of the response surface and helped in identifying the optimal conditions for maximum antimicrobial activity [72].

The sol–gel process was also optimized using DOE [73,74]. Sol–gel involves the transition of a system from a liquid “sol” into a solid “gel” phase. This method is widely used for producing coatings, fibers, and powders with controlled microstructure. DOE is instrumental in optimizing the various parameters involved in the sol–gel process. Key parameters that are used during the DOE optimization are those that significantly influence the sol–gel process and include the following: the type and concentration of precursors, pH, temperature, and aging time. By using a CCD design, we successfully identified which parameters significantly affect the properties of the final product and proved that, without efficient ultrasonic homogenization, there is limited possibility to functionalize the surface of the biodegradable polymers by antimicrobial coatings containing metal and metal oxide nanoparticles.

Once we defined the critical parameters, their optimal levels were determined by using RSM. For example, the mechanical properties and antimicrobial efficacy of the sol–gel coatings were optimized by adjusting the concentration of precursors and the processing conditions.

There are many excellent examples of optimization of polymer properties by different algorithms and, in the following subchapter, a case study on the optimization of preparation of antimicrobial polymer by the Design of Experiment statistical methodology will be presented. Design of Experiment (DOE) is a systematic methodology used to plan, conduct, analyze, and interpret controlled tests to evaluate the factors that may influence a particular process or product.

By using DOE, the relationships between various input variables and the resulting output, optimizing the process for better performance, quality, and efficiency are identified. This approach involves creating an experimental framework that considers all possible combinations of the factors being studied, which can be manipulated in a structured manner. Through statistical analysis, DOE helps in understanding the interactions between different variables, identifying significant factors, and determining the optimal conditions for desired outcomes. In fields such as materials science and polymer engineering, DOE is essential for accelerating development, reducing costs, and improving the reliability and robustness of products and processes [70,71,72,73,74,75,76,77,78,79].

When comparing DOE to MD, it can be seen that MD assists in predicting the dispersion and interaction of antimicrobial agents within nanocomposites, ensuring uniform distribution and enhanced properties. Recent scientific articles have thoroughly discussed MD simulations for predicting the dispersion and interaction of antimicrobial agents within nanocomposites. For example, Liu and Guo utilized MD simulations to understand how antimicrobial peptides interact with polymer nanocomposites, focusing on the dispersion and binding properties within the composite matrix [80]. This topic was also covered by Chen et al. (2020), who provided thorough insights into the dispersion and interaction of silver nanoparticles in polymer matrices using molecular dynamics simulations, highlighting the factors that influence the stability and antimicrobial effectiveness of the composites [81], as well as Su et al. (2020), who explored mechanical properties of pristine PE and PE/AgNP composites by molecular dynamics simulations [82].

Nanocomposite formation involves the integration of nanoparticles into a matrix material, typically polymers, ceramics, or metals, to enhance the composite’s properties. The resulting nanocomposites exhibit a unique combination of the matrix’s bulk properties and the nanoparticles’ functional properties, leading to materials with superior mechanical, thermal, electrical, and antimicrobial characteristics [83,84]. The formation process is crucial as it determines the dispersion, interaction, and overall performance of the nanocomposite. The nanoparticles important for the formation of composites include metal nanoparticles (e.g., silver, gold, copper), metal oxides (e.g., zinc oxide, titanium dioxide), and carbon-based materials (e.g., carbon nanotubes, graphene) [85]. These nanoparticles are selected based on the desired properties they impart, such as antimicrobial activity, electrical conductivity, or mechanical strength. Nanoparticles need to be preselected as they influence preparation, properties, and uses of polymer-layered silicate nanocomposites, emphasizing the incorporation of nanoparticles into polymers to enhance mechanical, thermal, and barrier properties [86,87]. In-depth exploration of the science and technology behind nanocomposites, including the methods for integrating nanoparticles into various matrices and the resulting effects on material performance are optimized by MD and ML methodologies.

As a matrix material, the most commonly used are polymers due to their versatility, ease of processing, and wide range of properties. Examples include polyethylene, polypropylene, polystyrene, and biopolymers like chitosan. Functionalization involves modifying the nanoparticle surface with chemical groups to improve compatibility and dispersion within the matrix. Proper dispersion of nanoparticles in the matrix is essential for achieving uniform properties throughout the nanocomposite. The nanocomposite is processed into the desired form using techniques such as extrusion, casting, injection molding, or 3D printing.

Singh and Sharma (2021) investigated the molecular interactions between antimicrobial agents and polymer nanocomposites, providing detailed MD simulation results on how these agents are dispersed and retained within the material [88], while Zhang et al. focused on the dispersion characteristics and mechanism of action of antimicrobial agents within nanocomposites, using MD simulations to model these complex interactions [89]. All those examples show how MD simulations are a necessary step in the efficient modeling and optimization of complex systems.

Processing conditions like temperature, pressure, and shear forces are optimized to maintain nanoparticle dispersion and achieve the desired properties. Achieving uniform dispersion of nanoparticles within the matrix is critical for optimizing the nanocomposite’s properties. Aggregation or poor dispersion can lead to weak points and reduced performance. Functionalization of nanoparticles and the use of surfactants or coupling agents can enhance dispersion. Strong interactions at the interface between nanoparticles and the matrix improve load transfer, mechanical properties, and overall stability. Chemical bonding, hydrogen bonding, and van der Waals forces can all contribute to these interactions. Therefore, the concentration of nanoparticles needs to be optimized. Too low a concentration may not impart the desired properties, while too high a concentration can lead to agglomeration and processing difficulties. Other processing conditions such as temperature, mixing speed, and processing time must also be carefully controlled to ensure proper dispersion and maintain the integrity of both the nanoparticles and the matrix.

Nanocomposites incorporating antimicrobial nanoparticles, such as silver or zinc oxide, are used in medical devices, wound dressings, food packaging, and water purification systems. These nanocomposites prevent microbial growth and enhance the longevity and safety of the products. Except for medicine applications, nanocomposites are used in automotive, aerospace, and construction industries for their superior mechanical properties, such as increased strength, stiffness, and resistance to wear and corrosion. Nanocomposites with conductive or semiconductive nanoparticles are used in flexible electronics, sensors, and photovoltaic cells due to their enhanced electrical and optical properties. Nanocomposites with nanoparticles like graphene or carbon nanotubes exhibit improved thermal conductivity and stability, making them suitable for thermal management applications in electronics and high-performance materials.

For all the above-mentioned applications, the formation of nanocomposites is a multifaceted process that requires careful consideration of the choice of nanoparticles and matrix, dispersion techniques, and processing conditions.

## 6. Case Study—Optimization of Preparation of Antimicrobial Polymer by Using Design of Experiments

In a practical application, DOE was used to optimize the formulation and processing parameters for a sol–gel coating intended for antimicrobial applications. The study involved screening of factors. By this initial screening experiment, the concentration of precursor, pH, and curing temperature had been identified as significant factors affecting the coating’s antimicrobial properties. RSM was then employed to optimize these factors. A CCD was used to model the response surface, and the optimal conditions were determined. The optimized conditions were validated experimentally, showing a significant improvement in antimicrobial activity without compromising the mechanical properties of the coating. According to the reported cases [70,71,72,73,74,75], the design of experiment was proven to be a versatile and powerful tool that enhances the efficiency and effectiveness of research in formulation, antimicrobial activity, and sol–gel processes. By systematically exploring the interactions between multiple variables, DOE enables researchers to develop optimized, high-performance materials and processes. The integration of DOE into experimental workflows not only accelerates the development cycle but also leads to more reliable and reproducible outcomes.

This short case study shows a total of 26 preliminary experiments were used to optimize the sol–gel process and to find the optimal combination of six process parameters aimed at achieving the best surface modification.

The sol–gel process was performed using the GLYMO (3-glycidyloxypropyltrimethoxysilane) as a precursor and 0.1 M HCl as a catalyst for creating an advanced antimicrobial surface, with tailored properties, on the polymer materials. GLYMO, an organosilane compound, acted as a network-forming agent that could be hydrolyzed and subsequently condensed to form a siloxane network. When HCl was used as a catalyst, it accelerated the hydrolysis and condensation reactions, facilitating the formation of a gel. The process began with the hydrolysis of the methoxy groups in GLYMO, producing silanol groups. These silanol groups then underwent condensation reactions, creating a three-dimensional siloxane network. The presence of HCl ensured a controlled reaction rate, which was crucial for achieving uniformity in the material’s microstructure. This sol–gel process was particularly valuable in the development of antimicrobial coatings, containing nanoparticles of silver and zinc oxide, where the incorporation of GLYMO enhanced properties such as adhesion, flexibility, and chemical resistance. The ability to fine-tune the process parameters allowed for the production of antimicrobial materials with enhanced mechanical and functional properties, making this process extremely efficient. In addition, the sol–gel process was intensified using an ultrasound for better homogenization of nanoparticles before application to the cellulose materials. A 0.1 M HCl solution was used as a catalyst as low concentration of strong acid make the sol–gel process much more efficient and robust. Higher concentrations would cause damage to the base materials (biodegradable polymers, in this case, the viscose surface). The decision on how to use relatively low acid concentrations was based on several factors, primarily that higher concentrations of acids and their mixtures destroy natural cotton fibers due to partial fiber degradation.

An output parameter that was taken as a goal of optimization was the recovery angle that is used to describe the flexibility of the polymer materials. The recovery angle is a key parameter in assessing the resilience and elasticity of textile materials. It measures the ability of a fabric to return to its original shape after being deformed, such as when it is stretched, bent, or compressed. This property is particularly important for applications where durability and shape retention are critical, such as in activewear, upholstery, and medical textiles.

The recovery angle is typically determined by a standardized test where a fabric sample is bent to a specified angle and then allowed to recover. The angle to which the fabric returns is measured and compared to its original state. A smaller recovery angle indicates better elasticity and shape retention, meaning the fabric can quickly and efficiently return to its original form. Conversely, a larger recovery angle suggests lower resilience and greater deformation under stress.

The recovery angle can be influenced by various factors, including the type of fibers used, the fabric weave or knit structure, and any finishing treatments applied. For example, fabrics treated with certain chemical finishes or those incorporating elastomeric fibers like spandex will generally exhibit superior recovery properties. Understanding and optimizing the recovery angle is essential for developing high-performance textiles that maintain their appearance and functionality over prolonged use and repeated mechanical stresses.

The determination of the recovery angle should not be confused with the determination of the contact angle, which is a critical parameter for evaluating the hydrophobic surface properties of polymer materials. The contact angle, defined as the angle formed at the junction of the liquid–solid interface, provides insight into the wettability and hydrophobicity of a textile surface. By measuring the contact angle, the material’s surface energy and its interaction with liquids are monitored, which are indicative of the material’s flexibility and performance characteristics. A lower contact angle suggests better wettability and flexibility, as the coating gel spreads more easily across the material surface, indicating a hydrophilic nature. Conversely, a higher contact angle indicates a hydrophobic surface with lower wettability, often correlating with less flexible materials. The measurement is typically performed using a goniometer, where a droplet of liquid (usually water) is placed on the surface and the angle formed at the liquid–solid–air interface is recorded. This method is essential for optimizing textile treatments, coatings, and finishes to achieve desired properties such as moisture management, stain resistance, and overall comfort. By understanding and manipulating the contact angle, manufacturers can enhance the functional performance and adaptability of textile materials to meet specific application requirements.

**Figure 5:**
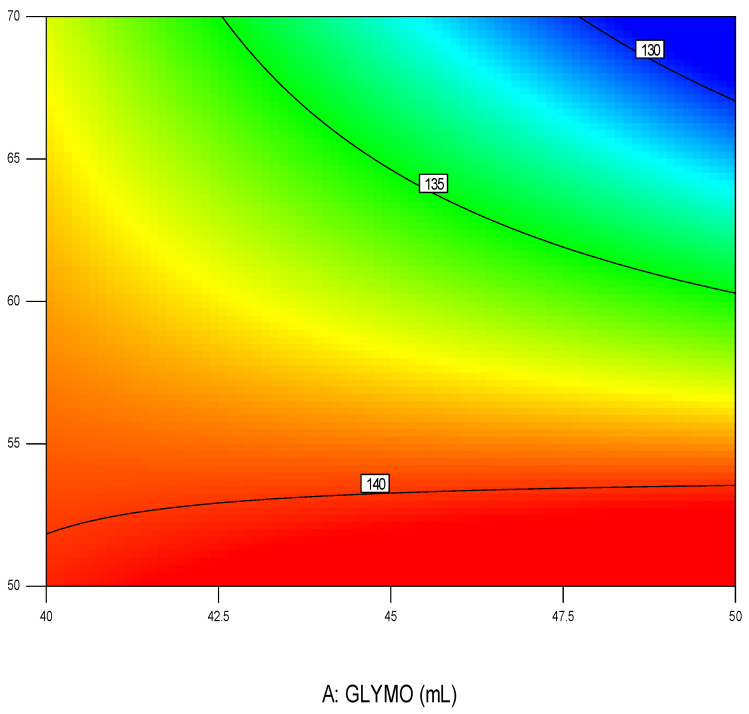
Graphical overview of modeled surface by using DOE methodology dependent on investigated process parameters GLYMO (from 40 to 50 mL in the process) and acid 01 M HCl catalyzator. The red color is the predicted maximal value that was the goal of the optimization, while the blue region describes the least wanted solution (combination of process parameters) since this would lead to the wors effects of the treatment.

The sol–gel procedure can significantly harden the surface of the previously flexible materials, which prevents usage of such materials on bandages and wound healing materials. Therefore, in this work, the surface modification which results in a maximally flexible yet efficient antimicrobial surface was a goal of optimization. The results of the obtained recovery angles are shown in Table 1. These results were recalculated as the average of three measurements (n = 3), and then optimized using software State Ease v. 9.01 by State Ease Company, USA. The obtained model is presented in Figure 5.

After performing preliminary experiments, a model was obtained, which correlates the desired flexibility with initial input parameters: Flexibility = 108.9 + 0.33 c(precursor), mgL^−1^ + 72.3 c(nanoparticles), % + 0.19 frequency(homogenization), Hz − 0.02 time, s. By this model, a correlation diagram was designed, as is presented in Figure 5.

**Figure 6:**
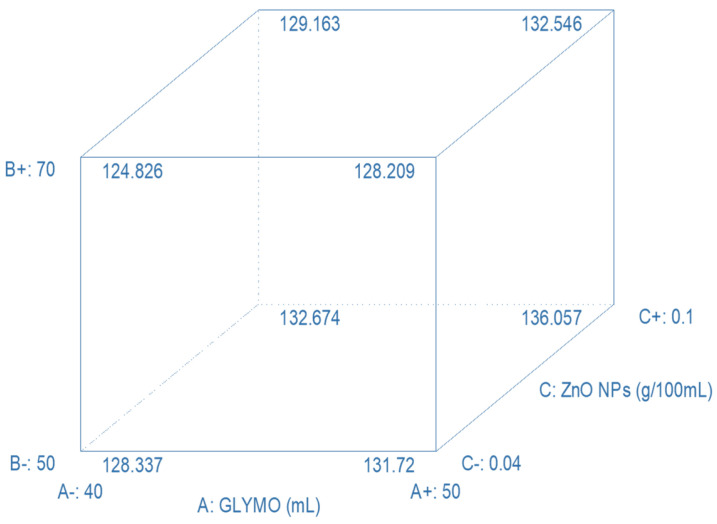
Experimental design of the process parameters in functionalization of polymer, modeling by design of experiment and optimization by response surface methodology, showing the constrains of each investigated parameter, e.g., GLYMO concentration from 40 to 50, concentration of ZnO colloid suspension of nanoparticles form 0.04 to 0.1 g/100 mL, and the concentration of the 0.1 M HCL acid catalyzator from 50 to 70 mL in a 1 L.

From the results in Figure 5, it can be seen that the most important parameter was the concentration of precursor and nanoparticles. After modeling, the next step is optimization, which involves finding the optimal value of a specific parameter, which in this case was the recovery angle. The process parameters and their constraints were selected and predefined before optimization (Figure 6).

The computer program offered many possible optimal combinations of process parameters that would result in maximum recovery angles. Two solutions from the proposed combinations were tested experimentally, and the obtained results were compared with the predicted values. The obtained relative error was very low, under 0.7%. This demonstrated that the model accurately describes the complex system of sol–gel modification of cellulose materials and can therefore be used to predict its behavior.

## 7. Discussion

The synergistic use of MD and ML represents a powerful paradigm in computational materials science and engineering. By combining the detailed atomistic insights provided by MD simulations with the predictive capabilities of ML algorithms, researchers can accelerate the design and optimization of complex materials, such as antimicrobial polymers and nanocomposites. This integrated approach leverages the strengths of both methodologies to overcome their individual limitations and unlock new possibilities in materials discovery and development.

How the integration of MD and ML provides a comprehensive approach to the design and optimization of antimicrobial materials was discussed in this paper.

The integration of computational methods in materials science, particularly for antimicrobial polymers and nanocomposites, presents several challenges and areas for future development. Addressing these challenges and exploring future directions will be crucial for advancing this field. Such methods are computationally intensive. MD simulations require significant resources to model complex molecular interactions over time, while deep learning models require substantial computational power and memory for training and inference. Additionally, integrating computational predictions with experimental validation is essential but challenging. Discrepancies between computational predictions and experimental results need to be resolved to refine models and improve accuracy. Therefore, for now, effective integration requires collaboration between computational scientists, experimentalists, and data scientists. Bridging the gap between these disciplines can be challenging due to differences in methodologies, terminologies, and objectives. Expanding the application of MD and ML to a broader range of antimicrobial materials, including novel polymers, peptides, and hybrid nanocomposites, will open new avenues for materials discovery and optimization.

The advantages of development of current technology enable a wide horizon of other methodologies that can be applied in materials science. For example, QM/MM (Quantum Mechanics/Molecular Mechanics) methodologies are powerful computational techniques used to study the properties and interactions of antimicrobial polymers at a molecular level [90]. These hybrid methods combine the accuracy of quantum mechanical calculations, which are essential for understanding electronic structures and reactions of antimicrobial agents, with the efficiency of molecular mechanics, which is suitable for simulating large polymer systems. This approach allows researchers to investigate the detailed mechanisms by which antimicrobial agents interact with polymer matrices, providing insights into the binding sites, stability, and reactivity of these compounds within the polymeric environment [91].

For antimicrobial polymers, QM/MM methodologies can elucidate how different antimicrobial agents, such as metal nanoparticles or organic molecules, integrate and interact within the polymer structure. This detailed understanding aids in optimizing the design of antimicrobial polymers to enhance their efficacy and stability. For instance, QM/MM simulations can reveal how the electronic properties of a metal nanoparticle are affected when embedded in a polymer matrix, or how an organic antimicrobial agent interacts with the polymer chains at an atomic level [92,93]. Such insights are crucial for designing polymers with improved antimicrobial activity and reduced resistance development.

Furthermore, QM/MM methods can help predict the physical and chemical properties of antimicrobial polymers, guiding experimental efforts and reducing the need for extensive trial-and-error approaches. By simulating different configurations and compositions, researchers can identify the most promising candidates for practical applications in medical devices, food packaging, and other areas where antimicrobial properties are essential. The combination of quantum and molecular mechanics thus provides a comprehensive toolkit for advancing the field of antimicrobial polymers, offering a pathway to more effective and durable antimicrobial solutions [93].

## 8. Conclusions

Computational methodologies, including MD, density functional theory, and ML, play a crucial role in the synthesis and application of antimicrobial polymers, biomolecules, and nanocomposites. These techniques enable efficient design, optimization, and understanding of material properties, paving the way for advanced antimicrobial materials with tailored properties. The integration of MD and ML holds great promise for advancing the field of antimicrobial materials. Addressing the challenges of computational cost, data quality, and the integration of experimental and computational methods will be crucial for realizing this potential. Future directions include developing more efficient algorithms, improving data integration, and expanding computational techniques to new antimicrobial systems. By fostering interdisciplinary collaboration and leveraging advanced computational methodologies, researchers can accelerate the discovery and optimization of innovative antimicrobial materials with tailored properties, paving the way for new applications in healthcare, environmental protection, and beyond.

## Figures and Tables

**Table 1:** Input and output parameters applied in optimization of polymer functionalization by antimicrobial nanoparticles.

	Concentration of Precursor mg/L	Nanoparticle Concentration %	Frequency of Homogenizator, Hz	Time, s	Obtained Flexibility, Angle °
1	45.0	0.70	67.5	2700	132.6
2	45.0	0.70	67.5	2700	126.6
3	45.0	0.70	67.5	2700	117.7
4	50.0	0.40	55.0	3600	121.7
5	35.9	0.70	67.5	2700	112.8
6	40.0	1.00	55.0	3600	130.7
7	45.0	0.70	67.5	1062	128.8
8	40.0	0.40	80.0	3600	129.3
9	45.0	0.70	44.7	2700	136.2
10	45.0	0.15	67.5	2700	129.2

## References

[1] Levy S.B., Marshall B. (2004). Antibacterial resistance worldwide: Causes, challenges and responses. Nat. Med..

[2] Cetin-Karaca H., Newman M.C. (2015). Antimicrobial efficacy of natural phenolic compounds against gram positive foodborne pathogens. J. Food Res..

[3] Shlaes D.M. (2015). Research and Development of Antibiotics: The Next Battleground. ACS Infect. Dis..

[4] Darouiche R.O. (2004). Treatment of infections associated with surgical implants. N. Engl. J. Med..

[5] Liu M., Bauman L., Nogueira C.L., Aucoin M.G., Anderson W.A., Zhao B. (2022). Antimicrobial polymeric composites for high-touch surfaces in healthcare applications. Curr. Opin. Biomed. Eng..

[6] Muñoz-Bonilla A., Fernández-García M. (2012). Polymeric materials with antimicrobial activity. Prog. Polym. Sci..

[7] Wang L., Hu C., Shao L. (2017). The antimicrobial activity of nanoparticles: Present situation and prospects for the future. Int. J. Nanomed..

[8] Lam S.J., Wong E.H., Boyer C., Qiao G.G. (2018). Antimicrobial polymeric nanoparticles. Prog. Polym. Sci..

[9] Lam S.J., O’Brien-Simpson N.M., Pantarat N., Sulistio A., Wong E.H., Chen Y.-Y., Lenzo J.C., Holden J.A., Blencowe A., Reynolds E.C. (2016). Combating multidrug-resistant Gram-negative bacteria with structurally nanoengineered antimicrobial peptide polymers. Nat. Microbiol..

[10] Aguilar Z. (2012). Nanomaterials for Medical Applications.

[11] Sadybekov A.V., Katritch V. (2023). Computational Approaches Streamlining Drug Discovery. Nature.

[12] Jiménez-Luna J., Grisoni F., Weskamp N., Schneider G. (2021). Artificial intelligence in drug discovery: Recent advances and future perspectives. Expert Opin. Drug Discov..

[13] Sabe V.T., Ntombela T., Jhamba L.A., Maguire G.E., Govender T., Naicker T., Kruger H.G. (2021). Current trends in computer aided drug design and a highlight of drugs discovered via computational techniques: A review. Eur. J. Med. Chem..

[14] Yu W., MacKerell A.D. (2017). Computer-aided drug design methods. Antibiotics.

[15] Gurung A.B., Ali M.A., Lee J., Farah M.A., Al-Anazi K.M. (2021). An Updated Review of Computer-Aided Drug Design and Its Application to COVID-19. BioMed Res. Int..

[16] Song C.M., Lim S.J., Tong J.C. (2009). Recent advances in computer-aided drug design. Brief. Bioinform..

[17] Zhong F., Xing J., Li X., Liu X., Fu Z., Xiong Z., Lu D., Wu X., Zhao J., Tan X. (2018). Artificial intelligence in drug design. Sci. China Life Sci..

[18] Hou T., Xu X. (2004). Recent development and application of virtual screening in drug discovery: An overview. Curr. Pharm. Des..

[19] Hill R.G., Richards D. (2021). Drug Discovery and Development E-Book: Technology in Transition.

[20] Durrant J.D., McCammon J.A. (2011). Molecular dynamics simulations and drug discovery. BMC Biol..

[21] Zhao H., Caflisch A. (2015). Molecular dynamics in drug design. Eur. J. Med. Chem..

[22] Huang D., Caflisch A. (2011). The free energy landscape of small molecule unbinding. PLoS Comput. Biol..

[23] Labanowski J.K., Andzelm J.W. (2012). Density Functional Methods in Chemistry.

[24] Banegas-Luna A.-J., Cerón-Carrasco J.P., Pérez-Sánchez H. (2018). A review of ligand-based virtual screening web tools and screening algorithms in large molecular databases in the age of big data. Future Med. Chem..

[25] Hasan A.S., Mohammed F.Q., Takz M.M. (2020). Design and synthesis of graphene oxide-based glass substrate and its antimicrobial activity against MDR Bacterial Pathogens. J. Mech. Eng. Res. Dev..

[26] Honarparvar B., Govender T., Maguire G.E., Soliman M.E., Kruger H.G. (2014). Integrated approach to structure-based enzymatic drug design: Molecular modeling, spectroscopy, and experimental bioactivity. Chem. Rev..

[27] Surabhi S., Singh B. (2018). Computer aided drug design: An overview. J. Drug Deliv. Ther..

[28] Kore P.P., Mutha M.M., Antre R.V., Oswal R.J., Kshirsagar S.S. (2012). Computer-aided drug design: An innovative tool for modeling. Open J. Med. Chem..

[29] Baig M.H., Ahmad K., Roy S., Ashraf J.M., Adil M., Siddiqui M.H., Khan S., Kamal M.A., Provazník I., Choi I. (2016). Computer aided drug design: Success and limitations. Curr. Pharm. Des..

[30] Sohraby F., Bagheri M., Aryapour H. (2019). Performing an in silico repurposing of existing drugs by combining virtual screening and molecular dynamics simulation. Computational Methods for Drug Repurposing.

[31] Lima A.N., Philot E.A., Trossini G.H.G., Scott L.P.B., Maltarollo V.G., Honorio K.M. (2016). Use of machine learning approaches for novel drug discovery. Expert Opin. Drug Discov..

[32] Coley C.W., Green W.H., Jensen K.F. (2018). Machine learning in computer-aided synthesis planning. Acc. Chem. Res..

[33] Klambauer G., Hochreiter S., Rarey M. (2019). Machine Learning in Drug Discovery. J. Chem. Inf. Model..

[34] Carpenter K.A., Huang X. (2018). Machine learning-based virtual screening and its applications to Alzheimer’s drug discovery: A review. Curr. Pharm. Des..

[35] Zhang L., Tan J., Han D., Zhu H. (2017). From machine learning to deep learning: Progress in machine intelligence for rational drug discovery. Drug Discov. Today.

[36] Bohr H. (2020). Drug discovery and molecular modeling using artificial intelligence. Artificial Intelligence in Healthcare.

[37] Krishnan S.R., Bung N., Bulusu G., Roy A. (2021). Accelerating de novo drug design against novel proteins using deep learning. J. Chem. Inf. Model..

[38] Gawehn E., Hiss J.A., Schneider G. (2016). Deep learning in drug discovery. Mol. Inform..

[39] Chen H., Engkvist O., Wang Y., Olivecrona M., Blaschke T. (2018). The rise of deep learning in drug discovery. Drug Discov. Today.

[40] Mendolia I., Contino S., Perricone U., Ardizzone E., Pirrone R. (2020). Convolutional architectures for virtual screening. BMC Bioinform..

[41] Gimeno A., Ojeda-Montes M.J., Tomás-Hernández S., Cereto-Massagué A., Beltrán-Debón R., Mulero M., Pujadas G., Garcia-Vallvé S. (2019). The light and dark sides of virtual screening: What is there to know?. Int. J. Mol. Sci..

[42] Liu X., Shi D., Zhou S., Liu H., Liu H., Yao X. (2018). Molecular dynamics simulations and novel drug discovery. Expert Opin. Drug Discov..

[43] Yan T., Yu L., Zhang N., Peng C., Su G., Jing Y., Zhang L., Wu T., Cheng J., Guo Q. (2022). The advanced development of molecular targeted therapy for hepatocellular carcinoma. Cancer Biol. Med..

[44] Chen X., Wang Y., Ma N., Tian J., Shao Y., Zhu B., Wong Y.K., Liang Z., Zou C., Wang J. (2020). Target identification of natural medicine with chemical proteomics approach: Probe synthesis, target fishing and protein identification. Signal Transduct. Target. Ther..

[45] Peters M.B., Raha K., Merz K. (2006). Quantum mechanics in structure-based drug design. Curr. Opin. Drug Discov. Dev..

[46] Hassan A.U., Sumrra S.H. (2022). Exploring the bioactive sites of new sulfonamide metal chelates for multi-drug resistance: An experimental versus theoretical design. J. Inorg. Organomet. Polym. Mater..

[47] Amusengeri A., Tata R.B., Bishop Ö.T. (2020). Understanding the pyrimethamine drug resistance mechanism via combined molecular dynamics and dynamic residue network analysis. Molecules.

[48] Vanommeslaeghe K., Guvench O. (2014). Molecular mechanics. Curr. Pharm. Des..

[49] Shoichet B.K. (2004). Virtual screening of chemical libraries. Nature.

[50] Sliwoski G., Kothiwale S., Meiler J., Lowe E.W. (2014). Computational methods in drug discovery. Pharmacol. Rev..

[51] Hoque I., Chatterjee A., Bhattacharya S., Biswas R. (2017). An approach of computer-aided drug design (CADD) tools for in silico pharmaceutical drug design and development. Int. J. Adv. Res. Biol. Sci..

[52] Dos Santos Nascimento I.J., De Aquino T.M., Da Silva-Júnior E.F. (2021). Drug repurposing: A strategy for discovering inhibitors against emerging viral infections. Curr. Med. Chem..

[53] Yadava U. (2018). Search algorithms and scoring methods in protein-ligand docking. Endocrinol. Metab. Int. J..

[54] Melo-Filho C.C., Braga R.C., Andrade C.H. (2014). 3D-QSAR approaches in drug design: Perspectives to generate reliable CoMFA models. Curr. Comput. Aided Drug Des..

[55] Verma J., Khedkar V.M., Coutinho E.C. (2010). 3D-QSAR in drug design—A review. Curr. Top. Med. Chem..

[56] Cherkasov A., Muratov E.N., Fourches D., Varnek A., Baskin I., Cronin M., Dearden J., Gramatica P., Martin Y.C., Todeschini R. (2014). QSAR modeling: Where have you been? Where are you going to?. J. Med. Chem..

[57] Patel H.M., Noolvi M.N., Sharma P., Jaiswal V., Bansal S., Lohan S., Kumar S.S., Abbot V., Dhiman S., Bhardwaj V. (2014). Quantitative structure–activity relationship (QSAR) studies as strategic approach in drug discovery. Med. Chem. Res..

[58] Wang M., Wang Z., Sun H., Wang J., Shen C., Weng G., Chai X., Li H., Cao D., Hou T. (2022). Deep learning approaches for de novo drug design: An overview. Curr. Opin. Struct. Biol..

[59] Chenthamarakshan V., Das P., Hoffman S., Strobelt H., Padhi I., Lim K.W., Hoover B., Manica M., Born J., Laino T. (2020). CogMol: Target-specific and selective drug design for COVID-19 using deep generative models. Adv. Neural Inf. Process. Syst..

[60] Schneider P., Walters W.P., Plowright A.T., Sieroka N., Listgarten J., Goodnow R.A., Fisher J., Jansen J.M., Duca J.S., Rush T.S. (2020). Rethinking drug design in the artificial intelligence era. Nat. Rev. Drug Discov..

[61] Rifaioglu A.S., Atas H., Martin M.J., Cetin-Atalay R., Atalay V., Doğan T. (2019). Recent applications of deep learning and machine intelligence on in silico drug discovery: Methods, tools and databases. Brief. Bioinform..

[62] Gilson M.K., Liu T., Baitaluk M., Nicola G., Hwang L., Chong J. (2016). BindingDB in 2015: A public database for medicinal chemistry, computational chemistry and systems pharmacology. Nucleic Acids Res..

[63] Mouchlis V.D., Afantitis A., Serra A., Fratello M., Papadiamantis A.G., Aidinis V., Lynch I., Greco D., Melagraki G. (2021). Advances in de novo drug design: From Conventional to Machine Learning Methods. Int. J. Mol. Sci..

[64] Arús-Pous J., Patronov A., Bjerrum E.J., Tyrchan C., Reymond J.-L., Chen H., Engkvist O. (2020). SMILES-based deep generative scaffold decorator for de-novo drug design. J. Cheminform..

[65] Liu X., IJzerman A.P., van Westen G.J. (2021). Computational approaches for de novo drug design: Past, present, and future. Artif. Neural Netw..

[66] Gentile F., Agrawal V., Hsing M., Ton A.-T., Ban F., Norinder U., Gleave M.E., Cherkasov A. (2020). Deep docking: A deep learning platform for augmentation of structure based drug discovery. ACS Cent. Sci..

[67] Chen H., Engkvist O. (2019). Has drug design augmented by artificial intelligence become a reality?. Trends Pharmacol. Sci..

[68] Klebe G. (2011). On the validity of popular assumptions in computational drug design. J. Cheminform..

[69] Renz P., Van Rompaey D., Wegner J.K., Hochreiter S., Klambauer G. (2019). On failure modes in molecule generation and optimization. Drug Discov. Today Technol..

[70] Rezić I., Rezić T., Bokić L.J. (2007). Optimization of the TLC separation of seven amino acids. J. Planar Chromatogr. Mod. TLC.

[71] Rezić I. (2011). Prediction of the surface tension of surfactants mixtures for detergent formulation using Design Expert software. Chem. Month..

[72] Rezić I. (2009). Optimization of ultrasonic extraction of 23 elements from cotton. Ultrason. Sonochemistry.

[73] Martinaga Pintarić L., Somogi Škoc M., Ljoljić Bilić V., Pokrovac I., Kosalec I., Rezić I. (2020). Synthesis, Modification and Characterization of Antimicrobial Textile Surface Containing ZnO Nanoparticles. Polymers.

[74] Rezić I., Kiš A. (2020). Design of Experiment Approach to Optimize Hydrophobic Fabric Treatments. Polymers.

[75] Rezić I., Majdak M., Ljoljić Bilić V., Pokrovac I., Martinaga L., Somogyi Škoc M., Kosalec I. (2021). Development of Antibacterial Protective Coatings Active Against MSSA and MRSA on Biodegradable Polymers. Polymers.

[76] Rezić I., Somogyi Škoc M., Majdak M., Jurić S., Sopko Stracenski K., Vinceković M. (2022). Functionalization of Polymer Surface with Antimicrobial Microcapsules. Polymers.

[77] Rezić I., Somogyi Škoc M., Majdak M., Jurić S., Sopko Stracenski K., Vlahoviček-Kahlina K., Vinceković M. (2022). ICP-MS Determination of Antimicrobial Metals in Microcapsules. Molecules.

[78] Vukoja D., Vlainić J., Ljoljić Bilić V., Martinaga L., Rezić I., Brlek Gorski D., Kosalec I. (2022). Innovative Insights into In Vitro Activity of Colloidal Platinum Nanoparticles against ESBL-Producing Strains of Escherichia coli and Klebsiella pneumoniae. Pharmaceutics.

[79] Rezić I., Kracher D., Oros D., Mujadžić S., Anđelini M., Kurtanjek Ž., Ludwig R., Rezić T. (2022). Application of Causality Modelling for Prediction of Molecular Properties for Textile Dyes Degradation by LPMO. Molecules.

[80] Liu Y., Guo M. (2019). Molecular Dynamics Simulation of Antimicrobial Peptides and Their Interactions with Polymer Nanocomposites. J. Phys. Chem. B.

[81] Chen X., Cheng Y., Huang J. (2020). Insights into the Dispersion and Interaction of Silver Nanoparticles in Polymer Matrices Using Molecular Dynamics Simulations. ACS Appl. Nano Mater..

[82] Su C.H., Chen H.L., Ju S.P., Chen H.Y., Shih C.W., Pan C.T., You T.D. (2020). The Mechanical Behaviors of Polyethylene/Silver Nanoparticle Composites: An Insight from Molecular Dynamics study. Sci. Rep..

[83] Hussain F., Hojjati M., Okamoto M., Gorga R.E. (2006). Polymer-matrix nanocomposites, processing, manufacturing, and application: An overview. J Comput. Mater..

[84] Ray S.S., Okamoto M. (2003). Polymer/layered silicate nanocomposites: A review from preparation to processing. Prog. Polym. Sci..

[85] Ajayan P.M., Schadler L.S., Braun P.V. (2003). Nanocomposite Science and Technology.

[86] Alexandre M., Dubois P. (2000). Polymer-layered silicate nanocomposites: Preparation, properties and uses of a new class of materials. Mater. Sci. Eng. R.

[87] Tjong S.C. (2006). Structural and mechanical properties of polymer nanocomposites. Mater. Sci. Eng. R.

[88] Singh S., Sharma P. (2021). Computational Modeling of Nanocomposite Systems for Antimicrobial Applications: A Molecular Dynamics Approach. J. Mater.Sci..

[89] Zhang W., Wang L., Zhao X. (2018). MD Simulation Study of the Interaction Mechanism between Antimicrobial Polymers and Bacterial Cell Membranes. Biomater. Sci..

[90] Senn H.M., Thiel W. (2009). QM/MM Methods for Biomolecular Systems. Angew. Chem. Int..

[91] Lin H., Truhlar D.G. (2007). QM/MM: What Have We Learned, Where Are We, and Where Do We Go from Here?. Theor. Chem. Acc..

[92] Chung L.W., Sameera W.M.C., Ramozzi R., Page A.J., Hatanaka M., Petrova G.P., Morokuma K. (2015). The ONIOM Method and Its Applications. Chem. Rev..

[93] Karelina M., Kulik H.J. (2017). Systematic Quantum Mechanical Region Determination in QM/MM Simulation. J. Chem. Theory Comput..

